# Comparison of Dynamic Hip Screw and Proximal Femoral Nailing Techniques in Stable Intertrochanteric Fractures

**DOI:** 10.7759/cureus.33366

**Published:** 2023-01-04

**Authors:** Salman Shiraz, Mohammad Shujauddin, Khalid Hasan, Ahmed Elramadi, Ghalib Ahmed

**Affiliations:** 1 Orthopaedics, Christine M. Kleinert Institute, Louisville, USA; 2 Orthopaedics Surgery, Hamad General Hospital, Doha, QAT; 3 Orthopaedics, Virginia Commonwealth University School of Medicine, Richmond, USA; 4 Orthopaedic Surgery, Hamad General Hospital, Doha, QAT; 5 Orthopedics, Hamad Medical Corpora, Doha, QAT

**Keywords:** postoperative outcomes, comparison between two surgical procedures, proximal femoral nail, dynamic hip screw fixation, stable intertrochanteric fracture

## Abstract

Introduction

Dynamic Hip Screw (DHS) and Proximal Femoral Nail (PFN) are two well-accepted modes of surgical treatments for intertrochanteric (IT) hip fractures. While studies have extensively explored the efficacy of one over the other in unstable fractures, the comparison is sparsely available for stable fractures. The main aim of this study is to compare DHS or PFN corrective surgeries in cases of stable IT fractures operated at the Hamad General Hospital, Doha, Qatar, between 2016 and 2018.

Methods

We conducted a retrospective data review of all stable IT fractures operated at the Hamad General Hospital, Doha, Qatar, between 2016 and 2018. Data were extracted from electronic medical CERNER records, including demographics, clinical notes, operative reports, radiographs, and imaging reports. Data review was followed by prospective data collection via phone about the current post-operative functional status of all cases. Data analysis was done on SPSS v.23. Study was approved by Medical Research Center (MRC) and Institutional Review Board (IRB) at Hamad Medical Corporation (HMC). Study Protocol ID: MRC-01-19-108

Results

Out of 62 stable IT fractures operated at our center during the study period, 42 underwent DHS correction, while 20 had PFN. The mean age of the studied cohort was 66.56 years (± 15.95). Males were twice more than females. The mean duration of surgery was 83.73 minutes for DHS and 120.25 minutes for PFN. This difference was statistically significant (p < 0.001). Differences in intraoperative blood loss, duration between fracture and surgery, and length of hospital stay were all statistically insignificant. Patients who underwent PFN showed a higher frequency of return to ambulation (assisted and unassisted), while the number of patients with DHS was less for the functional outcome. Similarly, more post-PFN radiographs displayed union than post-DHS radiographs (55% and 38%, respectively). This difference was statistically insignificant.

Conclusion

Our study showed promising results for stable IT fractures treated with PFN. However, more data and prospective observational studies are required to establish more statistically significant results.

## Introduction

Intertrochanteric (IT) hip fractures most commonly occur among post-menopausal females and the elderly [1-3]. IT fractures in younger age groups are most frequently caused by high-energy trauma (RTA) [4]. IT fractures significantly burden the global healthcare system due to longer life expectancies and a larger frequency of RTA [5]. Therefore, these fractures must be corrected as soon as possible.

A timely and appropriate surgical procedure guarantees a shorter post-operative hospital stay, reducing complications such as deep vein thrombosis (DVT), pulmonary embolism (PE), various heart or brain injuries, as well as the higher risk of contracting COVID-19 in the present [6].

Dynamic hip screws (DHS) and intramedullary nailing with proximal femoral nailing (PFN) are the two primary therapeutic methods that are frequently utilized to manage intertrochanteric fractures [7]. For stable intertrochanteric fractures, dynamic hip screw therapy is favored; intramedullary nailing is mostly used for relatively unstable fractures [8-10].

PFN has become a prominent therapeutic option for unstable IT fractures; however, comparing DHS and PFN in stable fractures has received scant attention in the literature [5,11]. This study analyzed the post-operative clinical and radiological results of stable intertrochanteric fractures treated at Hamad General Hospital in Doha, Qatar, using proximal femoral nailing or dynamic hip screw.

## Materials and methods

We retrospectively reviewed all of the stable intertrochanteric fractures that were operated on at the Hamad General Hospital in Doha, Qatar, between October 1, 2016, and September 30, 2018. The stability of the fracture was determined based on clinical notes and radiologic reports with clear documentation of intact posteromedial cortex [12]. The patients were contacted by phone to acquire prospective data regarding long-term post-surgical functionality and inquire about their ambulation status.

All adult patients (equal to or more than 18 years old at the time of injury) with stable fractures who were operated on during the study period were included. Polytrauma patients, patients less than 18 years at the time of injury, open fractures, unstable fractures, and cases where critical clinical and radiologic documentation was missing were excluded from the study.

Data including demographics, emergency or trauma room records, mechanism of injury, pre-operative orthopedic clinical notes, pre-operative radiologic images and reports, operative reports, intraoperative fluoroscopic interpretations, follow-up clinical notes, radiologic images, and reports were collected. The data was reviewed by the senior author (Senior consultant). Based on plain radiographs, the union was determined based on cortical bridging and fracture line disappearance.

Data was entered into password-protected Microsoft (MS) Excel files on password-protected PCs, with only the principal investigator having limited and allowed access. This ensured the secrecy of the data.

Analysis was performed on Statistical Package for Social Sciences version 23.0 for Windows (SPSS Inc., Chicago, IL). For categorical variables, frequency measures were used to summarize the data, and mean, and standard deviation (SD) were used for continuous variables. Statistical Package for Social Sciences version 23.0 for Windows will be used for the analysis (SPSS Inc., Chicago, IL). Paired T-tests were employed for parametric variables in the comparison between groups, while the Chi-square test was utilized for non-parametric variables. The ANOVA test was used to compare the variable mean values between the DHS and PFN groups. 

Ethical approval was obtained from the Medical Research Center (MRC) and Institutional Review Board (IRB) of Hamad Medical Corporation, Doha, Qatar. MRC# MRC-01-19-108.

## Results

Data from 62 patients who had corrective surgery for stable intertrochanteric fractures between 2016 and 2018 were examined. Out of these, 20 were treated with a proximal femoral nail and 42 with a dynamic hip screw. All the surgeries were performed either by a senior-level trainee (Final year resident) or a consultant. The responsible consultant was scrubbed and actively participated in/ supervised all cases.

In our study, the oldest participant was 93 years old, while the youngest was 25. For DHS and PFN, the average patient age is 66 years old. There were twice as many male patients (n=40 vs. n=22) as female patients (Table 1). We also noticed that low-velocity trauma, such as falls, caused frequent fractures (Table 2).

**Table 1:** Demographic data DHS: Dynamic hip screw, PFN: Proximal femoral nail, M: Male, F: Female

Observation	DHS	PFN	P Value
Mean Age Range	66.14	67.45	0.76
Gender (M: F)	1.80	1.86	-

**Table 2:** Mechanism of injury RTA: Road traffic accident

Mode of injury	Frequency	Percent
Unknown	2	3.2
Ground level fall	51	82.3
RTA	3	4.8
Fall from height	3	4.8
Tumor	2	3.2
Wrong movement	1	1.6

PFN had a little higher average intraoperative blood loss (180ml) than DHS (170ml). Compared to DHS, the average length of operation was longer in PFN (120.25 mins to 83.73 mins). In the DHS group, the surgery took less time overall, which was statistically significant (p < 0.001). The PFN group's average blood loss was slightly higher, and two patients needed blood transfusions after surgery. Although PFN recorded more blood loss, the DHS group had more instances requiring blood transfusions. (n=8, 19%) (Table 3).

**Table 3:** Comparison of observations between DHS and PFN-operated groups. DHS: Dynamic hip screw, PFN: Proximal femoral nail, min: Minutes, ml: Milliliters

Observation	DHS	PFN	P Value
Mean duration of surgery (mins)	83.73	120.25	0.001
Mean time of surgery after fracture (days)	1.88	2.75	0.21
Average blood loss (ml)	169	189	0.58
Patients requiring blood transfusion	8	2	-
Mean in-hospital stay (days)	9.71	12.10	0.38

In either group, there was no post-operative mortality. All patients' functional outcomes were evaluated at one-month, three-month, six-month, and yearly intervals. Better functional outcomes were observed after PFN, with a higher proportion of patients achieving ambulation (assisted and unassisted) compared to DHS, though this was not statistically significant (p-value> 0.05) (Table 4). Only two cases of bedridden patients underwent DHS after surgery and none after PFN.

**Table 4:** Functional Outcomes compared between DHS and PFN-operated groups *Adjusted for age, time to surgery from fracture, surgical complications, and comorbidities. A discrepancy in total observation is due to missing data. DHS: Dynamic hip screw, PFN: Proximal femoral nail

Functional Outcomes	DHS (n=32)*	PFN (n=16)*	P Value
				Bedridden	2	0	0.589
Wheelchair	8	2					
Ambulating with support	12	9					
Ambulating without support	10	5					

Radiological outcomes after PFN were better than DHS, with nearly a third more patients showing union on post-operative X-Rays, but the results were not statistically significant (p-value> 0.05) (Table 5).

**Table 5:** Radiological Outcomes compared between DHS and PFN-operated groups. *Adjusted for age, time to surgery from fracture, surgical complications, and comorbidities. A discrepancy in total observation is due to missing data. DHS: Dynamic hip screw, PFN: Proximal femoral nail

Radiological Outcomes	DHS (n=27)*	PFN (n=14)*	P Value
				Union	16	11	0.376
Non-union	11	3					

## Discussion

IT fracture treatment is heavily influenced by fracture type and bone quality. DHS was the preferred treatment modality for IT fractures until the last few decades when PFN was introduced [13]. Intramedullary devices, such as the PFN, were developed to address the shortcomings and complications associated with conventional extra-medullary devices, including non-union, re-operation rates, and malunion, particularly in unstable fractures [11,14,15]. Recent data show that intra-medullary devices can achieve union rates of up to 100% compared to extra-medullary devices [16]. Despite promising results with PFN, its overall efficacy in stable fractures remains contentious [5].

We compared the functional and radiological outcomes in stable IT fractures treated surgically with either PFN or DHS. For all cases, we also compared intraoperative blood loss (mL), the time between the fracture and surgery (in days), the length of post-operative in-hospital stay (in days), and surgery duration (in minutes).

Compared to the literature, our study found that the PFN procedure's mean duration was roughly 20 to 40 minutes longer than the DHS procedure. Longer time for patient positioning and prepping could be a contributor. Although the senior consultant responsible for the cases was always scrubbed, some of the surgeries were done by senior-level trainees, and the learning curve can contribute to increased time. Studies contrasting the results of PFN and DHS have largely demonstrated that the PFN procedure was quicker than DHS [5,17,18]. Few studies indicated that the lengths of the two procedures were comparable [19,20]. According to Das et al. analysis, PFN lasted longer when the nature of the fractures was more complicated [19].

The mean time between fracture occurrence and surgery was similar for both procedures. On average, patients treated with DHS received correction within two days of injury, while PFN was performed between 2 - 3 days. This may be a reason for pretty promising results from each technique, where almost 50 percent of the cases achieved full weight-bearing capacity postoperatively. The proportion of post-PFN return to ambulation is greater than DHS, as available in the literature [21]. While functional outcomes were better for PFN in the first three months, Myderrizi also noted in their work that there was no difference in recovery once the union was attained in six months [21]. As with our study, Zou et al. compared the two techniques and found no statistically significant differences in their functional outcomes [22]. In their prospective study of 135 patients, Baumgaertner et al. found similar functional recovery after either technique - however, they did demonstrate the superiority of the intramedullary nailing technique, particularly in unstable fractures [23]. Klinger et al. and Xu et al. reported immediate weight bearing on the first post-operative day after PFN [24, 25].

In comparison to DHS, we observed greater intraoperative blood loss during PFN. This is contrary to what was previously reported in the literature, where greater blood loss during DHS has been observed [22-26]. While eight patients from the DHS group required transfusions of up to 4 units in two cases, two patients who underwent PFN required blood transfusions of three units each.

Despite the difference in the proportion of patients with the observable union on radiographs, neither the PFN nor the DHS group had any malunions. Malunion has been noted in DHS cases in the literature [19].

Limitations

There are certain limitations in our study. Because the data were gathered retrospectively, their validity can be challenged, particularly regarding perioperative information like intraoperative blood loss. Additionally, because comments on radiological findings are often subjective, it can be challenging for the reviewer to draw reliable conclusions from the data. Study endpoints were not always accessible at the observed follow-up time points since the records were retrospective. Some data need to be included as a result of follow-up loss. We acknowledge a substantial loss to follow-up, including follow-up radiographs. The bulk of patients in the region is ex-pats. Many leave for their home countries after sustaining a significant injury like this, which was the main reason for discrepancies in the follow-up. By adding a prospective perspective to the study, we attempted to integrate the outcome findings simultaneously-however, the data needed to be improved because several patients were not available for responses.

## Conclusions

Our study showed promising results for stable IT fractures treated with PFN at our hospital. More scientific research is needed to treat stable intertrochanteric femur fractures. We suggest a prospective study comparing the two techniques be carried out on a larger cohort to establish statistically significant findings.
